# Effects of Soft Tissue Closure on Medication-Related Osteonecrosis of the Jaw in a Rabbit Model with Tooth Extraction: A Pilot Study

**DOI:** 10.1155/2021/4166770

**Published:** 2021-12-15

**Authors:** Ru Qing Yu, Jing Wen Li, Jing Yi Wang, Lei Huo, Li Wu Zheng

**Affiliations:** ^1^Discipline of Oral & Maxillofacial Surgery, Faculty of Dentistry, The University of Hong Kong, Hong Kong SAR, China; ^2^Department of Orthodontics, The First Affiliated Hospital of Zhengzhou University, Zhengzhou, Henan Province, China

## Abstract

**Objectives:**

The study investigated the effect of soft tissue closure after tooth extraction on the prevention of medication-related osteonecrosis of the jaw in a rabbit model.

**Materials and Methods:**

Twenty female New Zealand white rabbits were randomly assigned into the experimental group administrated with zoledronic acid (ZA) and control groups treated with saline. Bilateral lower premolar extraction was performed 4 weeks after ZA/saline administration. Immediately after extraction, the wound on the right mandible was closed by suture while the other side was left open. Animals were sacrificed 4 weeks and 8 weeks after tooth extraction. Fluorochrome labeling solutions were injected subcutaneously to evaluate the bone growth rates. The mandibles were harvested and subjected for microcomputed tomography, confocal microscope, and histomorphological examinations.

**Results:**

All extraction sites healed well without any signs of infection. Trabecular thickness (Tb.Th) was significantly higher in the ZA-treated group than in the control group at both week 4 and week 8, while no significant difference was detected in the rest of the assessed parameters. The bone growth rate in mandibles showed gradual reduction in the ZA-treated group. Histological analysis showed that at week 8, the animals in the ZA-treated group had significantly higher incidence of osteonecrosis than that in the control group, while no significance was revealed between the sutured and nonsutured side.

**Conclusions:**

ZA treatment significantly reduces bone growth rates but does not reveal a significant effect on bone mineral density and bone microarchitecture. Soft tissue closure of the extraction socket does not reduce the incidence of ONJ in the ZA-treated rabbit model.

## 1. Introduction

Bisphosphonates (BPs) are known to inhibit the activity of osteoclasts and thus reduce bone resorption and the subsequent bone remodeling. BPs are widely used against overactive osteoclasts in various bone diseases, such as osteoporosis, Paget's disease, and bone metastasizing malignant tumours, including multiple myeloma, breast cancer, and prostate cancer [1–3]. Medication-related osteonecrosis of the jaw (MRONJ) is a severe though rare complication after bisphosphonates and/or other antiresorptive or antiangiogenic medication treatment in a variety of malignant and benign bone diseases [4].

There are many risk factors associated with the development of MRONJ, including types, dosage, route of medication used, duration of therapy, dentoalveolar surgeries, age of the patients, systemic conditions, and genetic factors [4–7]. Among all the risks, the most common risk factor for MRONJ is tooth extraction [6, 8, 9].

MRONJ was described to occur exclusively in maxillofacial bones with a preference in mandible over maxilla [10–13]. One of the many explanations for this site-specific feature of MRONJ is that the oral microbes may cause inflammation or infection under the specific condition of oral wound [14–16]. However, how these oral microbes affect bone healing and whether an open oral wound puts an individual who is receiving IV BPs in a higher risk for developing osteonecrosis of the jaw remain unclear.

The objective of this study was to investigate the effect of soft tissue closure of tooth extraction on preventing MRONJ in a rabbit model treated with ZA.

## 2. Materials and Methods

### 2.1. Study Design

This study was approved by the Committee on Use Live Animal for Teaching and Research, The University of Hong Kong (CULATR No. 3774-15). The animal holding facility and the veterinary guidance were provided by Laboratory Animal Unite (LAU) of Li Ka Shing Faculty of Medicine, The University of Hong Kong.

Twenty adult female New Zealand white rabbits (3.2 kg to 4.2 kg) were randomly equally assigned into control and experiment groups. Five rabbits in each group were sacrificed at week 4 postoperatively as the short-term subgroups, and the other 5 rabbits were sacrificed at week 8 postoperatively as the long-term subgroups (the surgery day was set as the baseline of day 0). The rabbits in the experiment groups were given zoledronic acid subcutaneously three times per week with a dosage of 0.1 mg/kg for four weeks prior to surgery (rabbits underwent tooth extraction on mandible and dental implant placement on calvarial bone). The dental implant placement was for other investigation, and the results have been published [17]. The injection continued after the surgery and throughout the whole experiment until sacrifice. The control rabbits were given saline using the same regimen as that in the experimental group. In total, animals were injected 8 weeks and 12 weeks of zoledronic acid or saline in the short-term groups and long-term groups, respectively.

### 2.2. Tooth Extraction

All the tooth extraction procedures were performed under general anaesthesia. Heart rate, respiration rate, SpO_2_, and body temperature were monitored and recorded throughout the entire procedure. The rabbit was placed in a prone position with the head tilted slightly to the operating side. The gingival separator was used to separate the gingival tissue around the first premolar until touching the edge of the alveolar bone. Then, the root elevator was used to wedge between the root and alveolar bone lightly around the tooth and went slowly down as deep as possible. Lastly, the tooth was removed using a forcep. Tooth extraction was performed on the first premolars on both sides of the mandible. Curettage of the residual soft tissue in the tooth socket and irrigation of the surgical site were done subsequently. After confirming, there was no active bleeding of the wound, the wound of the extraction socket on the right side was sutured with a resorbable suture Vicryl (Ethilon Inc., Cornelia, GA), while the other side was left open. Postoperative analgesics and food care were provided under guidance. The food and water intake, body weight, behavior pattern, and daily activity were closely observed and recorded.

### 2.3. Fluorochrome Labeling

Three types of fluorochrome labeling solutions were prepared and were injected subcutaneously in a sequence using the protocol described in our recent publication [18, 19]. Briefly, calcein green (10 mg/kg, Sigma-Aldrich-C0875, St. Louis, USA), alizarin complexone (30 mg/kg, Sigma-Aldrich -A3882, St. Louis, USA), and oxytetracycline (20 mg/kg, Sigma-Aldrich -O5875, St. Louis, USA) with an interval of one week or two weeks, in the short-term subgroups and the long-term subgroups, respectively.

### 2.4. Sacrifice and Sample Collection

Euthanization of the animals was conducted by intravenous injection of ketamine through the ear vein at week 4 and week 8 postoperatively. The mandible was retrieved and preserved in 10% neutral buffered formalin solution for future assessment.

### 2.5. Microcomputed Tomography (Micro-CT) Examinations

**Figure 1:**
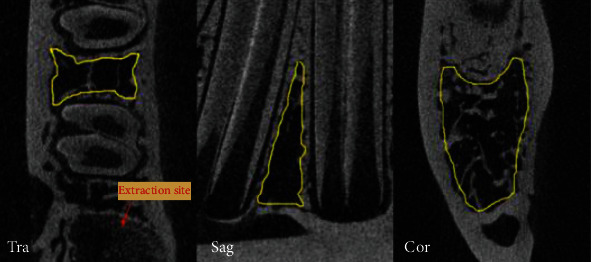
Region of interest (ROI), as outlined in the pictures, is selected at one tooth away from the extraction site. It is manually selected in the transverse view from the bottom layer (1^st^ slice) to the 300^th^ layer. The bottom layer is set at 100 slices above where the root was completely absent in the transverse view. The ROI is shown in three different planes here. Tra: transverse; Sag: sagittal; Cor: coronal.

For evaluation of the bone mineral density (BMD), bone volume fraction (BV/TV), and microtrabecular architecture, the specimens were scanned at 88 kV and 100 *μ*A intensity with a resolution of 17.3 *μ*m pixel using microcomputed tomography scanning (SkyScan1076; Bruker, Kontich, Belgium). The reconstruction data were retrieved and analyzed with the CT analyzer software, version 1.9 (Skyscan, Kontich, Belgium). The trabecular bone tissue in between two teeth at one tooth away from the extraction socket was manually selected as the region of interest (ROI) in the transverse view (Figure 1). The bottom layer was set at 100 layers above where the root was completely absent in the transverse view. In total, 300 layers were selected. For morphometric analysis of trabecular architecture, BV (bone volume), TV (tissue volume), BV/TV [20], Tb.Th (trabecular thickness, 3D measures of the average thickness of the cancellous bone structure [21]), Tb.N (trabecular number, the number of trabecular plates per unit length [20, 22]), and Tb.Sp (trabecular separation, average diameter of the marrow cavities [21]) were analyzed.

**Figure 2:**
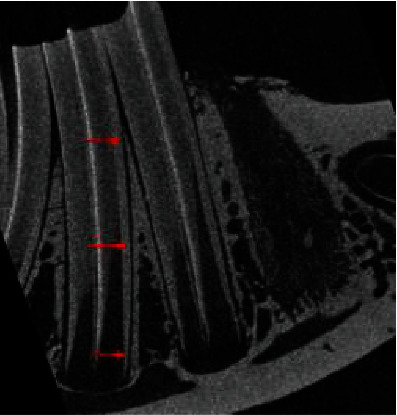
Periodontal ligament width is tested at 3 levels, neck, apical, and middle point between them. Both left and right first molars are measured.

In addition, the width of periodontal ligament space of the first molars was measured at three levels: tooth neck, middle, and apical (Figure 2). Data were summarized and subjected to statistical analysis.

### 2.6. Fluorochrome Labeling Analysis

After Micro-CT assessment, the undecalcified specimens were embedded in Technovit® 9100 PMMA (Heraeus Kulzer GmbH, Germany) and proceed to ground sections preparation for fluorochrome labeling and histological analysis. Each section was grinded and polished to approximately 30 *μ*m thickness. Each specimen was able to make 2 to 3 optimal sections.

Zeiss LSM 710 Upright Confocal Microscope and the Zeiss LSM 780 Inverted Confocal Microscope (Carl Zeiss, Oberkochen, Germany) were used for fluorochrome labeling analysis. The distances between two sequenced fluorochrome labeling were manually measured using the ZEN lite software at five randomly selected spot.

Bone growth rates were calculated by measuring the average distance between the two sequenced fluorescent line and then dividing the distance by the interval days between two sequenced injections according to the protocol of our previous study [19].

### 2.7. Histological Examinations

Sections were proceeded to histomorphological examinations after laser confocal imaging. Goldner's trichrome staining protocol was used to stain the sections [23].

Microscope images were obtained using the Eclipse LV100 POL (Nikon, Japan). Areas of osteonecrosis were defined by 8-10 adjacent empty osteocytic lacunae with the loss of osteocytes [24, 25]. Evaluation of osteonecrosis was determined as either no osteonecrosis or osteonecrosis presenting in the ten randomly selected high-power fields (20**×**), the incidence of which was calculated by dividing the number of animals with osteonecrosis by the total number of animals in a group at each time point.

### 2.8. Statistical Analysis

IBM SPSS statistics software (version 24.0, IBM Crop, Armonk:NY, USA) was for statistical analysis. Comparison of BMD, microstructure, bone growth rates, and incidence of osteonecrosis between groups was performed by independent *t*-test and Fisher's exact test at a significance level of 0.05.

## 3. Results

### 3.1. Clinical Observations

One rabbit in the ZA long-term group died one day after surgery, and the rest of the animals went through the whole experiment uneventfully. All extraction sites healed well without any signs of infection or inflammation (redness, swelling, etc.). No fistula or exposed bone was found.

### 3.2. Micro-CT Assessment

**Figure 3:**
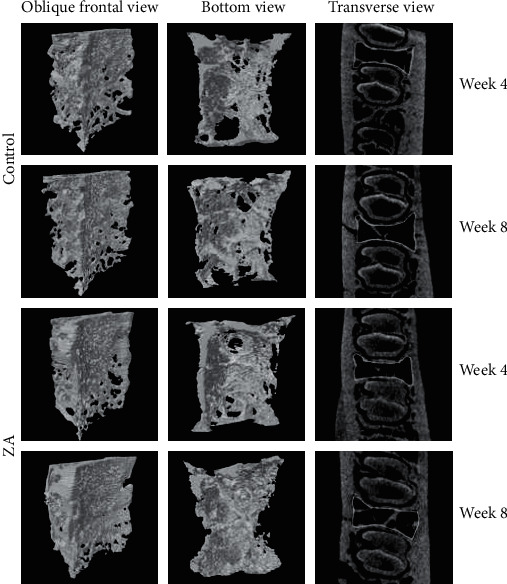
ROI demonstrated as three-dimensional models in oblique frontal view and bottom view. In transverse view also shows the ROI lined up in yellow.

**Figure 4:**
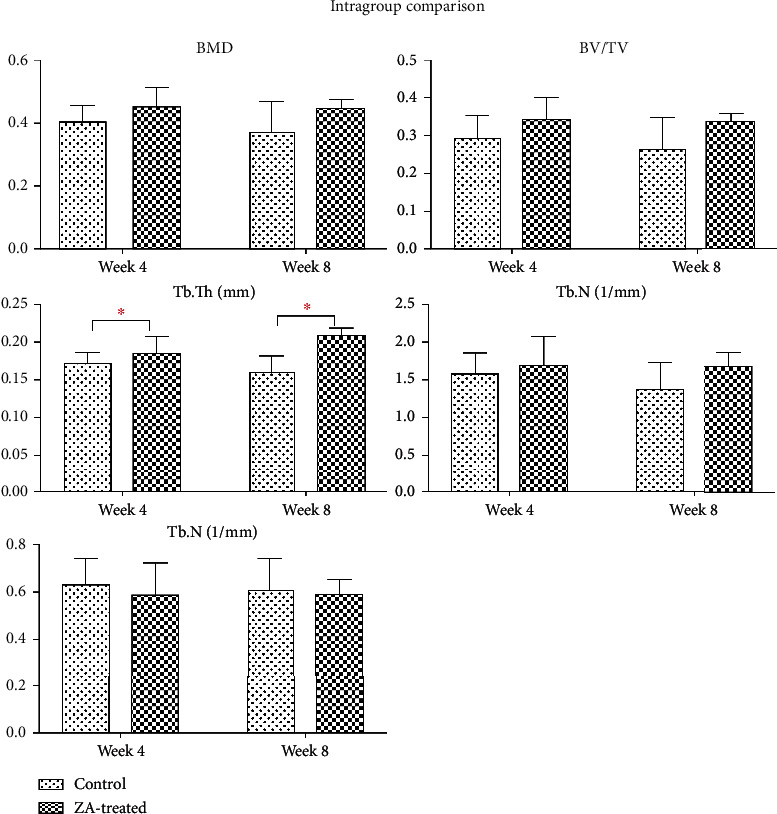
Intergroup comparison of BMD, BV/TV, Tb.Th, Tb.N, and Tb.Sp in the mandible between control and ZA-treated groups at time point of week 4 and week 8. Significant higher Tb.Th is seen in ZA-treated animals at both week 4 and week 8. No significant difference is detected in the rest of the assessed parameters.

**Figure 5:**
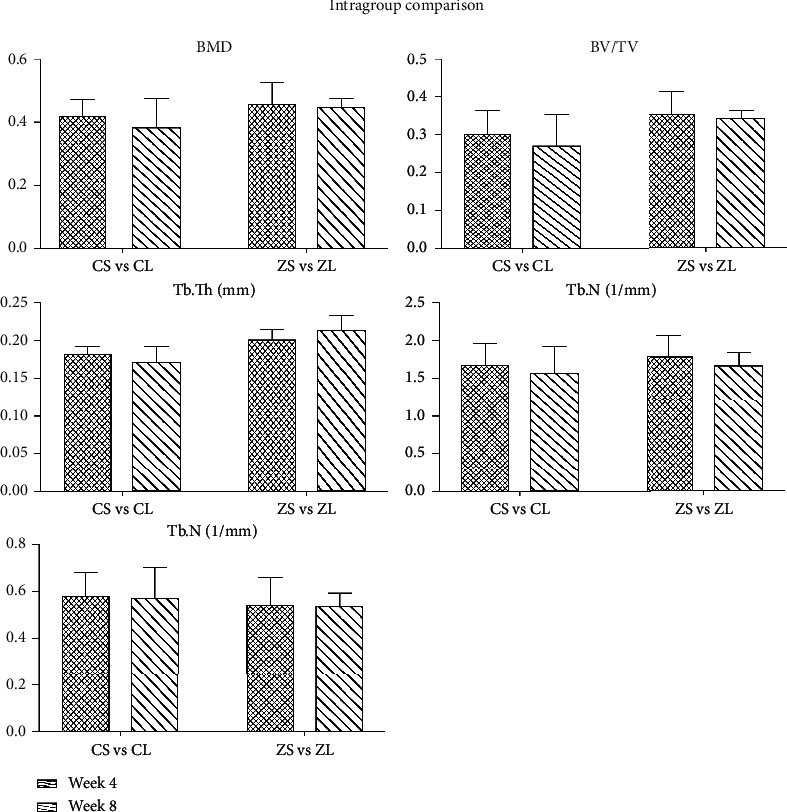
Intragroup comparison of BMD, BV/TV, Tb.Th, Tb.N, and Tb.Sp in the mandible between short-term subgroups (week 4) and long-term subgroups (week 8) in both control and ZA-treated groups. No significant difference is detected in any assessed parameters.

The reconstructed 3D images of ROI were shown in Figure 3. The results were displayed in the following Table 1 as mean ± SD. In the intergroup comparison, BMD, BV/TV, Tb.Th, and Tb.N in the ZA-treated group were higher, and Tb.Sp was lower than the control group at both week 4 and week 8 (Figure 4). However, the differences were not significant except for Tb.Th, which was significantly higher in the ZA-treated animals than that in the control animals at both week 4 and week 8. In the intragroup comparison, BMD, BV/TV, and Tb.N showed decrease at week 8 compared to those at week 4 in both control and ZA-treated group, only the reduction was less in the ZA-treated group. Tb.Th was higher at week 8 than that at week 4 in the ZA-treated group, and Tb.Sp barely changed from week 4 to week 8 in the ZA-treated group (Figure 5). The changing trends of Tb.Th in the ZA-treated group were opposite from those in the control group. However, the differences between each time point were not statistically significant in both control and ZA-treated groups.

**Figure 6:**
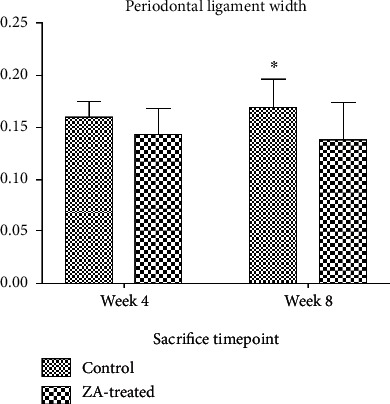
The ZA-treated groups showed thinner periodontal ligament space, more difference is shown in the comparison of control long-term group and ZA long-term group, but the difference is not statistically significant. Week 4, control short-term group, and ZA short-term group, which are sacrificed at week 4; week 8, control long-term group, and ZA long-term group, which are sacrificed at week 8. ∗Control long-term group, median is shown in the chart as the data in this group are not normally distributed.

The data of periodontal ligament space in the control long-term group were not normally distributed; therefore, Mann–Whitney test was used when comparison involved this group. Independent sample *t*-test was used in the other comparisons. Periodontal ligament space in the ZA treated groups, both long-term subgroup and short-term subgroup, was thinner than that of the respective control groups. More decrease was shown in the long-term subgroups compared to the short-term subgroups (Figure 6). However, the differences were not significant. When comparing the results of the control short-term group and the control long-term group, slight increase in periodontal ligament width was seen in the control long-term group. Slight decrease was shown when comparing the ZA short-term group and ZA long-term group. Still, neither of these differences was statistically significant.

**Figure 7:**
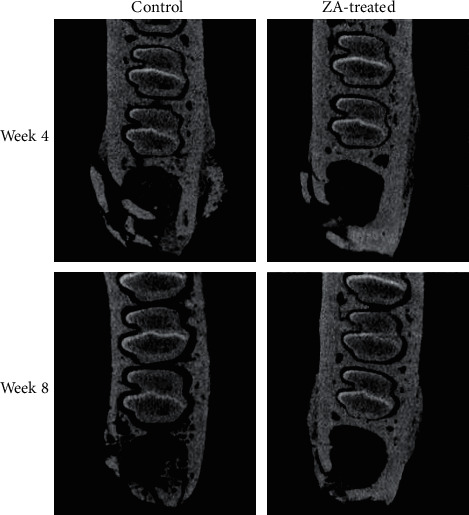
Micro-CT assessment of periosteal reaction after tooth extraction in the control and ZA groups at week 4 and 8.

**Figure 8:**
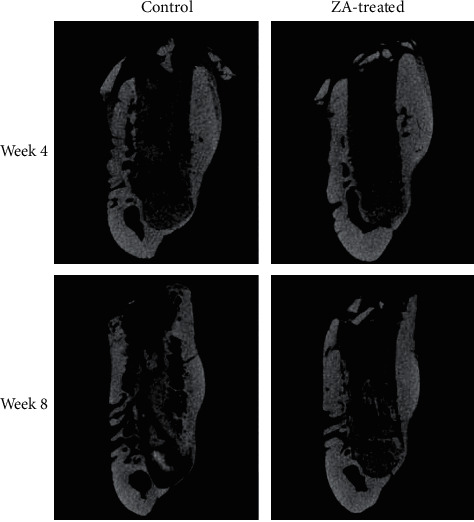
Micro-CT assessment of sequestra (coronal view) after tooth extraction in the control and ZA groups at week 4 and 8.

Periosteal reactions were seen in all groups except for in the control animals at week 8, as shown in Figure 7. Similarly, sequestra were seen in all groups except for in the control group at week 8 as well (Figure 8). Data were summarized in Table 2 describing the number of animals with periosteal reaction and sequestra formation in each group.

### 3.3. Fluorochrome Labeling Analysis

**Figure 9:**
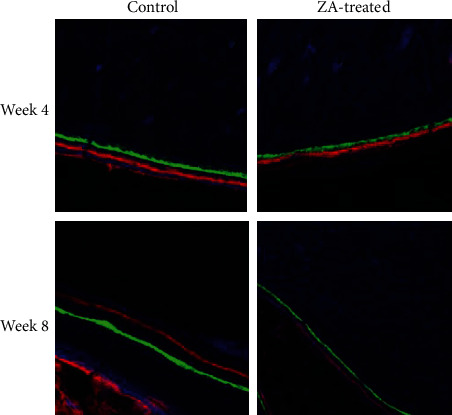
Fluorochrome labeling technique is used for evaluating the bone growth rates of mandibles.

**Figure 10:**
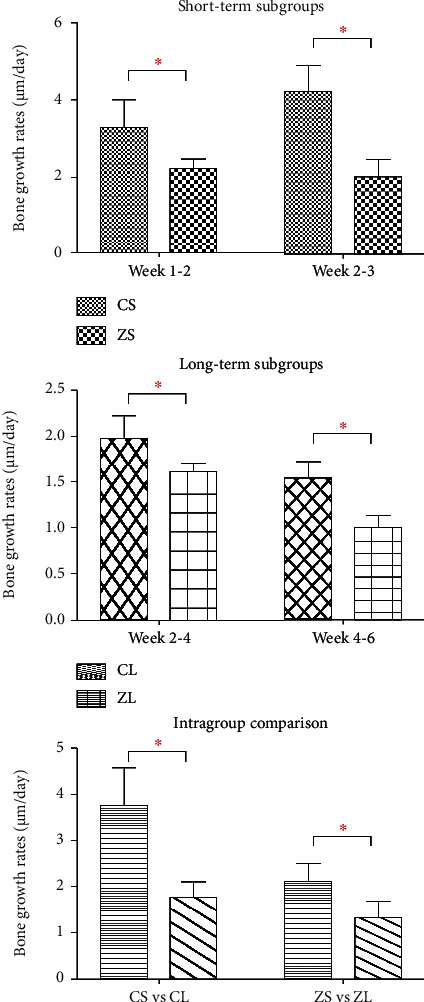
Intergroup comparison and intragroup comparison of bone growth rates (*μ*m/day) of the mandible. CS: control short-term subgroup; ZS: ZA short-term subgroup; CL: control long-term subgroup; ZL: ZA long-term subgroup. ∗Statistically significant.

The fluorochrome labeling images were shown in Figure 9. The results of the averaging bone growth rates were illustrated in Table 3. Both ZA-treated groups demonstrated significant reduced bone growth rates compared to control groups. In the intergroup comparison at week 4, the ZA-treated group showed significantly lower bone growth rates in both time periods than that of control counterparts. Similar results were detected at week 8 (Figure 10).

**Figure 11:**
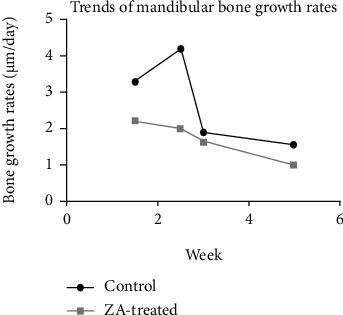
The rate of bone growth in mandibles shows gradual reduction in the ZA-treated group. While in the control group, a sharp increase is seen at week 2-3, following by a remarkable and then slow decrease.

Bone growth rates dropped markedly in week 4-6 compared to those in week 2-4 in the long-term subgroups. Similarly, bone growth rates reduced significantly in week 2-3 in comparison with those in week 1-2 in the short-term subgroups. After averaging the bone growth rates of different time periods in each subgroup, intragroup comparison was conducted in both control groups and experiment groups separately. Significant differences were detected in both intragroup comparisons. Remarkable lower bone growth rates were demonstrated in both long-term subgroups (Figure 10). When merging the data of the short-term subgroup and the long-term subgroup, as illustrated in Figure 11, the rate of bone growth in mandibles showed gradual reduction in the ZA-treated group. While in the control group, a sharp increase was seen at week 2-3, following by a remarkable and then slow decrease.

### 3.4. Histomorphological Analysis

One out of five rabbits (20%) in the control group was found with histological osteonecrosis defined as 8-10 adjacent empty osteocytic lacunae with the loss of osteocytes at week 4, while none was discovered at week 8. In the ZA-treated group, three out of five (60%) animals revealed histological osteonecrosis at week 4, while three out of four (75%) animals were found with histological osteonecrosis at week 8. The one histological osteonecrosis in the control group was found on the left side of the mandible which was the unsutured side. The sites of the osteonecrosis and the statistical analysis were summarized in Table 4. The one histological osteonecrosis in the control group was found on the left side of the mandible which was the unsutured side. Among the three histological osteonecrosis lesions in experimental group at week 4, two of them were on the sutured side. Similar results were seen in those at week 8, and two of the three identified histological osteonecrosis were on the sutured side. Fisher's exact test was performed to evaluate the differences in the incidence of histological osteonecrosis between control and experimental groups at each time point. Intragroup comparison between two time points and between left and right side was also conducted using the Fisher's exact test. Significant higher incidence (75%) of histological incidence was found in the ZA-treated group (0%) compared to the control group at week 8 (*P* = 0.048). However, no significant difference was detected in all the other comparisons.

## 4. Discussion

The position paper of the American Association of Oral and Maxillofacial Surgeons (AAOMS) has proposed the diagnostic criteria of MRONJ as current or precursory use of antiresorptive and antiangiogenic agents, nonhealing bone exposure or probable fistula in the maxillofacial area for at least 8 weeks without a history of radiation exposure [4]. Some studies suggested radiographic signs of osteonecrosis alone would be enough to diagnose MRONJ [26, 27], though not widely accepted. Bianchi et al. [28] found that computed tomography (CT) was very sensitive for abnormalities in the jaw bones and provide good accuracy in the outline of the pathologies in about 94% of the patients detected. In the same study, dental panoramic radiograph was demonstrated to be of limited use in assessing ONJ lesions. Some studies reported that magnetic resonance imaging (MRI) and CT scan were useful investigations in defining osteonecrotic lesions [29]. In accordance with these findings, Raje et al. [30] described bone sclerosis and fragmentation, periosteal new bone formation, and a big sequestrum in one case in the CT scans, therefore, concluded that radiographic findings were reasonably accurate in advanced, clinically established ONJ. However, the competence of CT for early detection of ONJ lesions remains unclear.

Radiographic findings of ONJ generally include osteosclerosis, osteolysis, a thickened lamina dura, periosteal bone deposition/proliferation, and sequestra formation [30, 31]. In our study, periosteal reaction and sequestra were observed in the micro-CT scanning. Four out of five animals in the control group exhibited periosteal reaction at week 4 while none did at week 8. In the ZA group, all five animals at week 4 and three out of four of those at week 8 showed periosteal reaction. As for sequestra formation, one out of five control rabbits were detected at week 4 compared to none found at week 8; in contrast, four out of five rabbits were found with sequestra formation in the ZA group at week 4 while one out four were identified at week 8. More cases of sequestra formation were found in ZA-treated animals compared with their control counterparts. While most animals exhibited periosteal reaction except in the control long-term subgroup. This was further confirmed by the histological examination that no periosteal bone deposition was observed in the control long-term group. In addition, there was no histological osteonecrosis detected in this subgroup.

Periosteal reaction may be caused by different insults, for instance, trauma, infection, and tumor [32]. The periosteal reaction observed in this study did not seem to relate to ZA treatments, in other words, the periosteal reaction was not the presentation of osteonecrosis lesions. In fact, it may be more reasonable to relate the periosteal reaction to the trauma caused during the extraction procedure.

In the micro-CT assessment, BMD, BV/TV, Tb.Th, Tb.N, and Tb.Sp demonstrated expected results in the ZA group when compared to the control group, though the differences in all parameters were not significant. The similar nonsignificant difference was also reported in other studies in healthy animals [33, 34]. However, this nonsignificant result may also be due to the small sample size in the study.

In this study, bone growth rates showed remarkable reduction in all time points in the ZA group when compared to the control group. ZA is a potent bisphosphonate that work mainly by inhibiting the osteoclasts' activity and then decreasing the bone turnover rates. The bone formation decrease was thought to follow the reduced bone resorption and the subsequent reduced bone remodeling rates [35]. The significant decrease in bone growth rates may explain why BMD and other microarchitecture parameters showed only slight increase or even decrease when a large dose of ZA was used and therefore the consequent expected strong inhibitory effect on bone resorption.

The main microscopic characteristics of MRONJ are necrotic bony trabeculae with empty osteocytic lacunae [36]. Commonly, the necrotic bone is enclosed by colonies of bacteria and demonstrates irregular resorption and notable reversal lines [37].

Areas of empty osteocytic lacunae (8-10 adjoining ones) with the loss of osteocytes were defined as the histological osteonecrosis in this study. When assessing the incidence of histological osteonecrosis, ZA-treated animals showed significantly higher incidence of histological osteonecrosis than the control counterparts at week 8. However, the incidence of histological osteonecrosis between the left and right side of the mandibles in both control and experimental groups exhibited no difference. Moreover, no exposed bone or probable fistula was identified in all animals. Therefore, it seemed that closing the extraction socket after tooth extraction did not have significant effect on the bone healing or the development of osteonecrosis.

Infection and inflammation have been proposed as a possible pathophysiology of MRONJ because this condition occurred exclusively to the jaw bones which are protected by a thin epithelium from the open oral cavity, which contains over 700 different species of microbes [38]. The jaw bones could be easily exposed to the bacteria after tooth extraction, trauma, or through the inflammatory dental diseases. The existence of some microorganisms that are distinct to the oral cavity has been found to be associated with the initiation or the progress of MRONJ. Cultures and biopsy from MRONJ patients have identified the presence of microbes including *Actinomyces*, *Fusobacterium*, *Staphylococcus*, *Eikenella*, *Bacillus*, and *Streptococcus* [39–43].

Clinical observation in this study exhibited no delayed wound healing on either the unsutured side or the sutured side in all animals. Moreover, histological assessment revealed no significant difference in the occurrence of histological osteonecrosis between the suture side and the open wound side. Indeed, an open wound would heal uneventfully even with the presence of normal oral microbes usually. However, the bone healing was hypothesized to be impeded with bisphosphonates treatment due to the decreased bone resorption, which makes it difficult for the inflammatory cells to arrive at pathogens and thus the inflammation and/or infection might progress [44]. The cumulated bacterial toxins and the hyperoxide generated in the inflammation response will contribute to the development of bone necrosis [44]. This was supported by some animal experiments showing that inflammation or infection could promote osteonecrosis of the jaw in those treated with antiresorptive medications [45–50]. Furthermore, mucoperiosteal coverage after tooth extraction may reduce the risk for ONJ in a bisphosphonate-treated rat model [51].

Different from the theoretical hypothesis, our results showed that whether to close the extraction socket or not did not appear to affect the bone healing or the development of ONJ. Unlike mucoperiosteal coverage, simple closure of mucosa after tooth extraction seemed not to be an effective way to reduce the development of ONJ in the rabbit model. One potential issue which may affect the result is the use of prophylaxis antibiotics which lowers the chance of infection and thus reduces the effect of soft tissue closure on preventing potential infection.

In conclusion, bisphosphonates significantly reduce bone growth rates but do not reveal a significant effect on bone mineral density (BMD) and bone microarchitecture; Soft tissue closure of the extraction socket does not reduce the incidence of ONJ in bisphosphonates-treated rabbits. Future study with larger sample size and no prophylaxis antibiotics to investigate the effect of different surgical modification on the prevention of MRONJ shall be taken into consideration.

## Figures and Tables

**Table 1:** Statistical analysis result of bone mineral density (BMD) and bone microstructure indices (mean ± SD).

Parameters	Groups					
	Control long-term	Control short-term	ZA long-term	ZA short-term	*P* value∗	*P* value∗∗
BV/TV	0.27 ± 0.08	0.30 ± 0.06	0.34 ± 0.02	0.35 ± 0.06	0.118	0.248
BMD	0.38 ± 0.09	0.41 ± 0.06	0.45 ± 0.02	0.46 ± 0.06	0.137	0.259
Tb.Th (mm)	0.17 ± 0.02	0.18 ± 0.01	0.21 ± 0.02	0.20 ± 0.01	0.012	0.019
Tb.N (1/mm)	1.53 ± 0.36	1.65 ± 0.27	1.63 ± 0.18	1.75 ± 0.27	0.638	0.582
Tb.Sp (mm)	0.59 ± 0.11	0.58 ± 0.11	0.55 ± 0.06	0.55 ± 0.11	0.546	0.722

BV/TV: bone volume/tissue volume; BMD: bone mineral density; Tb.Sp: trabecular separation; Tb.Th: trabecular thickness; Tb.N: trabecular number. ∗*P* value between control group and ZA group at week 8. ∗∗*P* value between control group and ZA group at week 4.

**Table 2:** Radiographic and histologic observations.

Group	Week	No.	Micro-CT		Histology	
			Sequestra	Periosteal reaction	Sequestra	Histological osteonecrosis
Control	4	5	1	4	3	2
	8	5	0	0	0	0

ZA	4	5	4	5	5	4
	8	4	1	3	4	4

**Table 3:** Bone growth rates measured by fluorochrome labeling.

Time	Bone growth rates (*μ*m/day)			*t*-test
	Groups	*N*	Mean ± SD	Sig. (2-tailed)^∗^
Week 1-2	CS	5	3.32 ± 0.64	0.016
	ZS	5	2.24 ± 0.22	

Week 2-3	CS	5	4.21 ± 0.68	0.000
	ZS	5	2.02 ± 0.45	

Week 2-4	CL	5	2.00 ± 0.24	0.024
	ZL	4	1.64 ± 0.09	

Week 4-6	CL	5	1.56 ± 0.19	0.002
	ZL	4	1.05 ± 0.11	

CS: control short-term group; ZS: ZA short-term group; CL: control long-term group; ZL: ZA long-term group. ∗*P* value.

**Table 4:** The incidence of osteonecrosis of the control and experimental group on the mandible at week 4 and week 8.

Groups	Control		ZA-treated		*P* value∗	*P* value∗∗
Time point	Week 4	Week 8	Week 4	Week 8		
ONJ	2 (L∗1, R∗1)	0	4 (L∗2, R∗3)	4 (L∗2, R∗2)		
NON-ONJ	3	5	1	0		
*N*	5	5	5	4		
Incidence	40%	0%	80%	100%	0.524	0.008
*P* value∗∗∗	—	—	1	—		
*P* value∗∗∗∗	0.444		1			

∗Intergroup comparison of the incidence between control and ZA-treated groups at week 4; ∗∗intergroup comparison of the incidence between control and ZA-treated groups at week 8, significant difference in the incidence of histological osteonecrosis was detected between control and ZA-treated group; ∗∗∗intragroup comparison of the incidence at each time point in each group between left and right sides; ∗∗∗∗intragroup comparison in the control and ZA-treated group.

## Data Availability

The data used to support the findings of this study are included within the article.
